# Endoscopic Ultrasound-Guided Radiofrequency Ablation of the Pancreatic Tumors: A Promising Tool in Management of Pancreatic Tumors

**DOI:** 10.1155/2016/4189358

**Published:** 2016-07-10

**Authors:** Kinesh Changela, Rashmee Patil, Sushil Duddempudi, Vinaya Gaduputi

**Affiliations:** ^1^Department of Gastroenterology, The Brooklyn Hospital Center-Clinical Affiliate of Mount Sinai Hospital, Brooklyn, NY 11201, USA; ^2^Department of Internal Medicine, Mount Sinai West, New York, NY, USA; ^3^Department of Gastroenterology, St. Barnabas Hospital, Bronx, NY, USA

## Abstract

*Objective*. Radiofrequency ablation is a well-established antitumor treatment and is recognized as one of the least invasive therapeutic modalities for pancreatic neoplasm. Endoscopic ultrasound-guided radiofrequency ablation (EUS-RFA) delivery can be used to treat both pancreatic cancer and asymptomatic premalignant pancreatic neoplasms and may serve as a less invasive alternative to surgical resection. This is an appealing option that may result in less morbidity and mortality. The aim of this review was to summarize and evaluate the clinical and technical effectiveness of EUS-guided RFA of pancreatic neoplasms.* Methods*. A through literature review was performed to identify the studies describing this novel technique. In this review article, we have summarized human case series. The indications, techniques, limitations, and complications reported are discussed.* Results*. A total of six studies were included. Overall, a 100% technical success rate was reported in human studies. Complications related to endoscopic ultrasound-guided radiofrequency ablation delivery have been described; however, few cases have presented life-threatening outcomes.* Conclusion*. We believe that this novel technique can be a safe and effective alternative approach in the management of selected patients.

## 1. Introduction

Pancreatic cancer carries a poor prognosis, with a 5-year overall survival rate of <5% and a median survival of <6 months. Though resection provides a chance for cure in some cases and increases life expectancy, only one-fifth of patients present with resectable disease. Established treatment modalities such as chemotherapy or chemoradiation therapy are options for patients with pancreatic cancer; however, they do little for overall outcomes [1, 2]. New modalities, such as radiofrequency ablation (RFA), are described in literature for the treatment of pancreatic cancer. Radiofrequency ablation (RFA) is a well-established antitumor treatment and is recognized as one of the least invasive therapeutic options for pancreatic cancer. RFA works by emitting energy resulting in coagulative necrosis of the surrounding tissue [3]. RFA is considered a safe and potentially curative method and has been used widely for the treatment of tumors of the liver, lung, and kidney but not for the treatment of the pancreas. The reluctance of clinicians to use RFA for pancreatic cancer may be related to the fear of adverse events, such as thermal injury-induced pancreatitis, thermal damage to structures around the pancreas (stomach, duodenum, portal vein, superior mesenteric vessels, and bile duct), and technical limitations. Furthermore, pancreatic cancer usually has diffuse margins making it difficult to ablate completely with just one procedure [4–7].

Recent studies have shown that RFA is feasible in patients with unresectable pancreatic cancer in an open, laparoscopic, or percutaneous setting. The delivery of ablative agents and devices to localized malignancies has become increasingly possible through a number of developments. Particularly, EUS-guided RFA (EUS-RFA) allows real-time imaging of the pancreatic neoplasm, where RFA may result in safe tissue ablation. EUS-RFA has been described by using a modified EUS needle and a commercial RF needle. RFA provides localized tissue ablation that ranges from 1 to 3 cm from the needle catheter [8–10]. For pancreatic EUS-RFA, several studies have reported the feasibility and safety of the procedure in animals. Overall, these studies have concluded that EUS-RFA of the pancreatic head with either monopolar or bipolar probes was well tolerated in the porcine pancreas and resulted in a minimal amount of pancreatitis. The studies also reiterated that the procedure was technically feasible, effective, and relatively safe in these animal models [4, 7, 9].

More recently, the feasibility and safety of EUS-RFA have been described in human studies. This review article will summarize the results of these studies.

## 2. Materials and Methods

An extensive English language literature search was conducted using PubMed, Medline, and Google to identify peer-reviewed original and review articles using the keywords “endoscopic ultrasound,” “radiofrequency ablation,” “pancreas,” “solid tumor,” and “EUS-RFA.” Human articles were selected. The references of pertinent studies were manually searched to identify additional relevant studies. The indications, procedural details, technical and clinical success rates, complications, and limitations were considered as part of the inclusion criteria. Search results yielded mostly small sample sized prospective studies and retrospective studies which limited statistical analysis in the form of meta-analysis.

## 3. Results

Six original articles published were considered appropriate to be included in the review article. All were human case series from India [12], UK [8, 13], South Korea [10], and China [14] or a case report from Italy [11]. All studies have been summarized in Tables 1 and 2.

### 3.1. Demographics

All studies were human studies. In these studies, a total of 28 patients were included. 18/28 (64%) patients were male and 10/28 (36%) were female. The average age of patients included was 61 years (see Table 1).

### 3.2. Indications

In all human studies, patients included presented with a pancreatic lesion. 17/28 (61%) of patients had proven adenocarcinoma of the pancreas. 6/28 (21%) were found to have a pancreatic neuroendocrine tumor and three of these patients included in the study by Lakhtakia et al. had a symptomatic insulinoma. Other pathologies of the pancreas included a mucinous cyst and a microcystic adenoma. The average size of the lesion in human studies was 37.8 mm.

### 3.3. Technique

**Figure 1:**
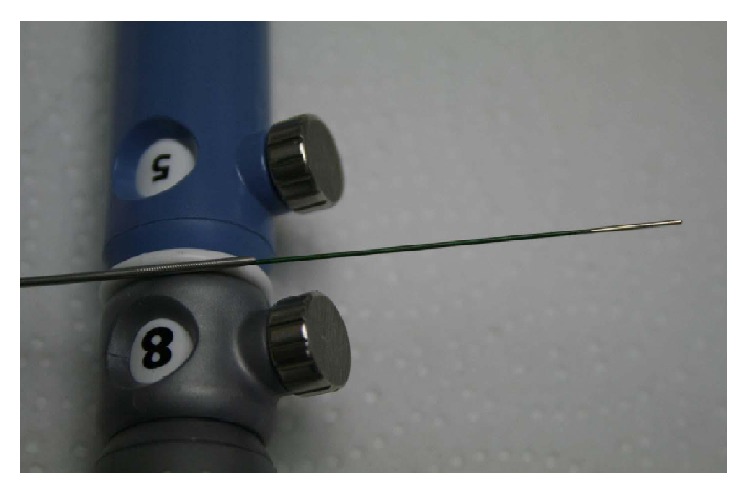
Habib*™* EUS-RFA probe (reproduced from manufacturer's website with appropriate permission [4]).

**Figure 2:**
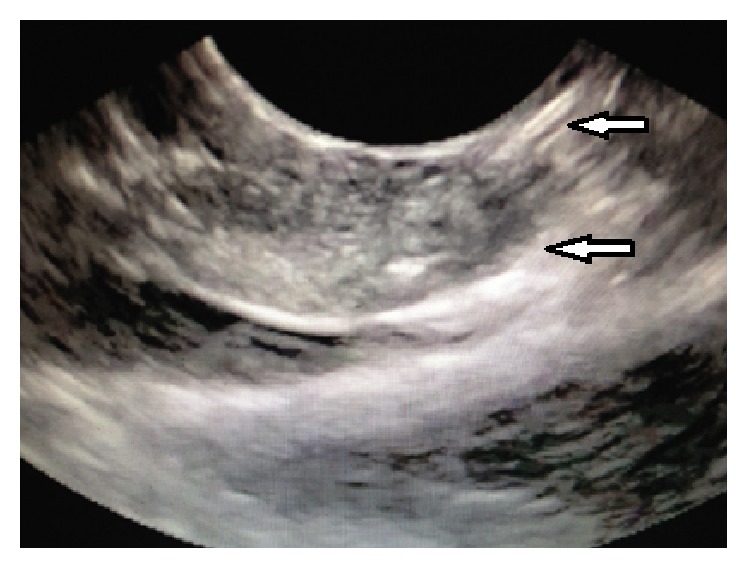
Endoscopic ultrasound view showing EUS-RFA probe inserted into the porcine pancreas (the porcine pancreatic tissue was ablated with RFA after placing EUS-guided 19-gauge Wilson Cook needle into the pancreas via transduodenal approach) (reproduced from an open access article under terms of the Creative Commons Attribution License [4]).

In all studies included in the review article, RF catheter was utilized to deliver the EUS-RFA. A summary of details of the techniques used in human versus animal studies is given in Table 2. In 4/6 (67%) of the studies, the novel Habib catheter [Habib EUS-RFA catheter, EMcision Ltd., London] was placed through a 19-gauge or 22-gauge fine needle aspiration (FNA) needle. The Habib EUS-RFA is a 1 Fr wire that can be inserted through the biopsy channel of an echoendoscope (Figure 1). To coagulate tissue in the pancreas, RF power is applied to the electrode at the end of the wire. Though our studies focused on EUS-RFA in the pancreas, the same modality could also be used in the liver. The Habib EUS-RFA is a monopolar device and is used in conjunction with a patient grounding/diathermy pad. This novel system comes in dispensing sheath and the catheter is removed from the dispensing sheath and connected to the adaptor cable, which is then connected to the generator. Power in the generator is set to the required wattage and a patient grounding/diathermy pad is applied as close to the operating field as possible, since the catheter is monopolar. Once the catheter is placed through EUS control and by using a 19-gauge biopsy needle with a stylet, RF energy is then applied for 90–120 s in most cases at the set wattage (Figure 2). In larger lesions, the Habib EUS-RFA probe and needle are pulled back as one unit and repositioned to ablate the lesion. This process can be repeated as many times as needed to ensure complete ablation of the lesion [12–14].

Another device that was utilized in Carrara et al. was a new flexible bipolar hybrid ablation device (ERBE Elektromedizin, Tübingen, Germany). This hybrid cryotherm probe (CTP) combined bipolar RF ablation with cryotechnology. A bipolar system ablates with less collateral thermal damage than a monopolar system but with less efficiency overall. The cryotechnology in this probe is used both to combine the advantages of the two approaches and to overcome the disadvantage of less efficiency: the more effective cooling by cryogenic gas increases the RF-induced effects [7]. Studies not using the Habib EUS-RFA or ERBE utilized the VIVA RF generator (STARmed, Koyang, Korea). This device utilized an 18 G FNA needle to introduce the catheter but otherwise had a similar approach to the Habib EUS-RFA catheter.

### 3.4. Technical and Clinical Success Rate

**Figure 3:**
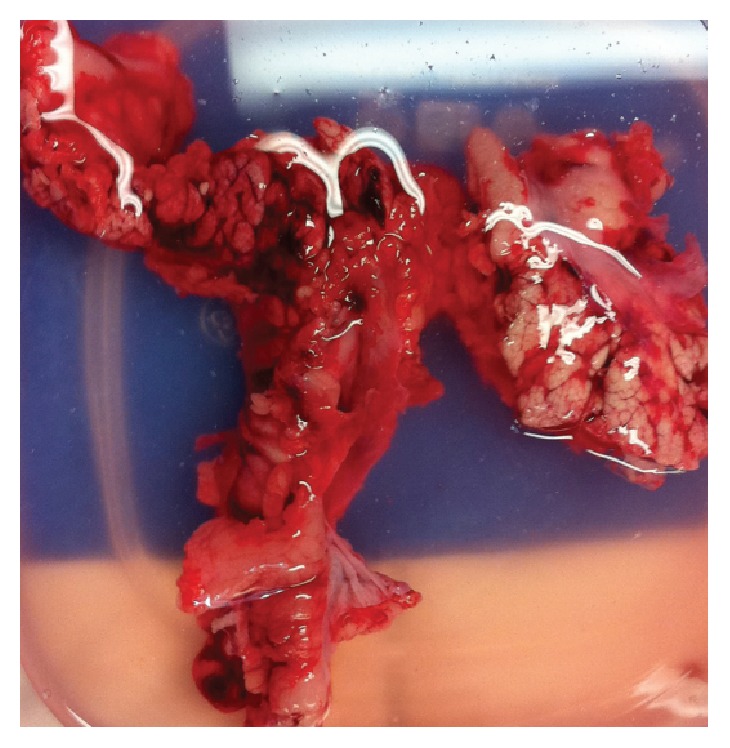
Pathological specimen of porcine pancreas (on the 6th day after procedure, the pigs were euthanized. The pancreas of the pigs was immediately excised surgically for gross examination of damage and tissue response) (reproduced from an open access article under terms of the Creative Commons Attribution License [4]).

Technical success was reported as 100% in human studies. In Lakhtakia et al. EUS-RFA proved effective for symptom relief in symptomatic pancreatic insulinoma for 3/3 (100%) of patients. In Pai et al. [8] the response ranged from complete resolution of the pancreatic lesion to a 50% reduction in the diameter of the lesion.  Figure 3 shows pathological specimen of porcine pancreas revealing tissue response after pigs were euthanized. In Wang et al. EUS-RFA of pancreatic carcinoma was technically easy and safe and well tolerated by the patients and achieved a considerable reduction in tumor size and CA19-9 levels. In animal studies, clinical success rates were measured more by complications and adverse outcome rates of the procedure.

### 3.5. Complications and Adverse Outcomes

In all six human studies, no major clinical complications or adverse outcomes were reported. Pai et al. [8] and Song et al. reported mild abdominal pain in 25% and 33% of patients, respectively. Pai et al. [13] reported a case of mild pancreatitis in 1 out of 7 patients (14%). In animal studies that have been reported in the literature but not included in our tables, clinical complications were reported postmortem when assessing pancreatic tissue and other surrounding structures. In Gaidhane et al., 2/5 (40%) of pigs had moderate pancreatitis, while in Carrara et al., one pig was reported to have necrotic pancreatitis with peritonitis. Another common complication reported in animal studies was fibrosis and adhesions.

### 3.6. Limitations

Thus far, clinically successful cases have been published with few complications reported, but this may be due to a publication bias as the procedure is fairly new. As more cases that are technically and clinically relevant are published, further data may be assessed regarding the potential efficacy and safety of EUS-RFA in the treatment of pancreatic tumors.

## 4. Summary and Future Directions

RFA is a well-recognized, safe, and effective modality for the treatment of pancreatic neoplasms, including unresectable pancreatic carcinoma. The technique is minimally invasive and has very good tolerability. EUS-RFA can be used to treat premalignant, asymptomatic, pancreatic lesions instead of surgical resection, and this is promising as surgical procedures are commonly associated with major morbidity and some mortality. Despite surgical and oncological advances in the treatment of pancreatic cancer, the prognosis remains poor partially because only 10% of patients have a chance to receive curative surgery [8]. EUS-RFA, as an alternative to surgery, is a well-established antitumor treatment using local thermal-induced coagulative necrosis [1]. In our review of the 6 articles published in the literature, technical success was reported as 100% in human studies. EUS-RFA provided effective symptomatic relief in patients with pancreatic lesions and significantly reduced the tumor size in the patients included in these studies.

Though EUS-RFA is technically successful in many cases, there have been clinical complications associated with the technique. The pancreas is a very thermosensitive organ, and when heat is applied on the normal pancreas, it produces an inflammatory response causing edema and later fibrosis and occasionally cystic transformation. In general, adverse events are more associated with the duration of ablation. Using lower energy may allow for multiple ablations with lower morbidity [2–5]. Adverse events related to EUS-RFA may include acute pancreatitis, pancreatic leaks, infection of necrotic pancreatic tissue, and posttreatment bleeding. Furthermore, it should be considered that the pancreas is different from other organs like the liver and RFA protocol for the liver cannot be used in the pancreas; the optimal thermal kinetic characteristics for the pancreas have not been determined, so there is still no standardized protocol for pancreatic RFA. The pancreas is also surrounded by many vital structures and pancreatic RFA has a risk of thermal injury to these surrounding organs including vasculatures. Finally, it is difficult to ablate pancreatic cancer completely and two or more procedures may be necessary in many cases [7–9]. In our review article, no major clinical complications or adverse outcomes were reported in human subjects. Pai et al. [8] and Song et al. reported mild abdominal pain in 25% and 33% of patients, respectively. Pai et al. [13] reported a case of mild pancreatitis in 1 out of 7 patients (14%). In animal studies, however, more serious complications such as necrotic pancreatitis with peritonitis and fibrosis and adhesions causing obstruction have been reported [4, 7]. Compared to studies on intraoperative RFA, most studies of EUS-RFA are animal studies testing for feasibility, safety, and efficacy of the procedure. In the studies included in our review, the authors concluded that EUS-RFA was feasible and safe in vivo in most cases, though occasionally more serious side effects did occur.

Until now, the feasibility and safety of EUS-RFA for the treatment of advanced pancreatic cancer and other pancreatic lesions have not been addressed. As the results of our review article have shown, EUS-RFA can be a technically feasible and safe option for patients with unresectable pancreatic cancer and may serve as an adjunct to treatment methods for unresectable disease. These preliminary data results compiled from our review suggest that the procedure is technically easy and safe. The responses to EUS-FNA ranged from complete resolution of a pancreatic lesion to a 50% reduction in diameter to symptomatic relief in patients and animal subjects. Further multicenter experience is required to identify its yield and safety in different stages of pancreatic cancer. In summary, we believe that further prospective studies are necessary to demonstrate the overall survival benefit of EUS-RFA for pancreatic cancer before widespread use of this novel procedure.

## Figures and Tables

**Table 1:** Characteristics of studies describing endoscopic ultrasound-guided radiofrequency ablation (EUS-RFA).

Study, location	Total subjects	Sex, male/female	Age	Study type
Armellini et al., Italy [11]	1	1/0	76	Case report
Lakhtakia et al., India [12]	3 patients	3/0	45	Case series
Pai et al., UK [8]	8 patients	7/1	65	Case series
Song et al., South Korea [10]	6 patients	1/5	62	Case series
Pai et al., UK [13]	7 patients	4/3	69	Case series
Wang et al., China [14]	3 patients	2/1	63	Case series

N/A: not applicable.

**Table 2:** Summary of reports describing endoscopic ultrasound-guided radiofrequency ablation (EUS-RFA).

Study, location	Diagnosis	Average size of lesion (mm)	Approach	RFA equipment	Needle size	Strength of RFA	Number of RFA sessions (average)	Technical success	Complications	Follow-up (months)	Comments
Armellini et al., Italy [11]	PNET	20	NM	VIVA RF generator (STARmed, Koyang, Korea)	18 G	5 W	1	1/1 (100%)	None	1	This report adds to the increasing evidence of PNETs being successfully treated by ablative therapies, which may represent a potential alternative to surgery in selected cases.

Lakhtakia et al., India [12]	PNET (insulinoma) = 3	NM	TG = 2 TD = 1	Novel internally cooled needle electrode	19 G	NM	NM	3/3 (100%)	None	5	EUS-RFA is feasible, apparently safe, and effective for symptom relief in symptomatic pancreatic insulinoma.

Pai et al., UK [8]	Mucinous cyst = 4 IPMN = 1 Microcystic adenoma = 1 PNET = 2	36.5	TG = 8	Habib EUS-RFA catheter	19 G or 22 G	5 W = 3 15 W = 2 20 W = 2 25 W = 1	4.5 (2–7)	8/8 (100%)	2/8 (25%) had mild abdominal pain	3–6	The response ranged from complete resolution to a 50% reduction in diameter of lesion.

Song et al., South Korea [10]	Pancreatic cancer in head = 2 or in body = 4	38 (30–90)	NM	VIVA RF generator (STARmed, Koyang, Korea)	18 G	20 W or 50 W	1.3 (1-2)	6/6 (100%)	2/6 (33%) had mild abdominal pain	2–6	EUS-RFA may be used as an adjunct and effective alternative treatment method for unresectable pancreatic cancer.

Pai et al., UK [13]	Pancreatic ductal adenocarcinoma = 7	35.2	NM	Habib EUS-RFA catheter	19 G or 22 G	5 W = 1 10 W = 3 15 W = 3	3 (2–4)	7/7 (100%)	1/7 (14%) had mild pancreatitis	3–6	

Wang et al., China [14]	Pancreatic carcinoma	37.3	NM	Habib EUS-RFA catheter	22 G	10 W or 15 W	3.7	3/3 (100%)	None	1.5	EUS-RFA of pancreatic carcinoma was technically easy and safe and well tolerated by the patients and achieved a considerable reduction in tumor size and CA19-9 levels.
